# Therapeutic Time Window With DWI-ADC (Diffusion-Weighted Imaging-Apparent Diffusion Coefficient) Match and Periodic Discharges for Status Epilepticus

**DOI:** 10.7759/cureus.53811

**Published:** 2024-02-07

**Authors:** Daiki Gohara, Shuichiro Neshige, Hideaki Sakahara, Narumi Ohno, Hirofumi Maruyama

**Affiliations:** 1 Department of Clinical Neuroscience and Therapeutics, Hiroshima University Hospital, Hiroshima, JPN; 2 Department of Clinical Neuroscience and Therapeutics, Hiroshima University, Hiroshima, JPN; 3 Department of Clinical Neuroscience and Therapeutics, Hiroshima University Graduate School of Biomedical and Health Sciences, Hiroshima, JPN

**Keywords:** status epileptics, partial epilepsy, electroencephalography (eeg), ct and mri brain, seizure medications

## Abstract

A man in his 70s with alcoholic dementia was admitted for acute, prolonged impaired consciousness. Blood and cerebrospinal fluid findings were unremarkable. Brain MRI revealed multiple high-signal cortical regions. Following diazepam and levetiracetam administration, electroencephalography (EEG) revealed <1 Hz lateralized periodic discharges, indicating that the seizures were ceasing. The periodic discharges had disappeared during the gradual recovery process by day 10; however, cortical arterial spin labeling findings persisted only in regions exhibiting cytotoxic edema. Without additional anti-seizure medication, no seizure recurred, but cognitive dysfunction remained. He was transferred to a rehabilitation hospital with the continued oral administration of levetiracetam at 1,000 mg/day. DWI-ADC (diffusion-weighted imaging-apparent diffusion coefficient) match may suggest an indication of a missed suitable treatment window for seizures.

## Introduction

Status epilepticus, arising from epileptic seizures, is a neurological emergency, underscoring the urgency for clinicians to control seizures to minimize secondary brain damage [1]. While MRI scans with arterial spin labeling (ASL) are valuable for screening not only stroke [2] but also seizures [3], their effectiveness is constrained by the low temporal resolution when used as an evaluation method to establish a diagnosis of status epilepticus. In contrast, critical care electroencephalography (EEG) is crucial for capturing dynamic changes over time in status epilepticus. Therefore, an essential component involves a combined evaluation of EEG and MRI. We herein present a case of status epilepticus assessed through the time course of EEG and MRI.

## Case presentation

**Figure 1:**
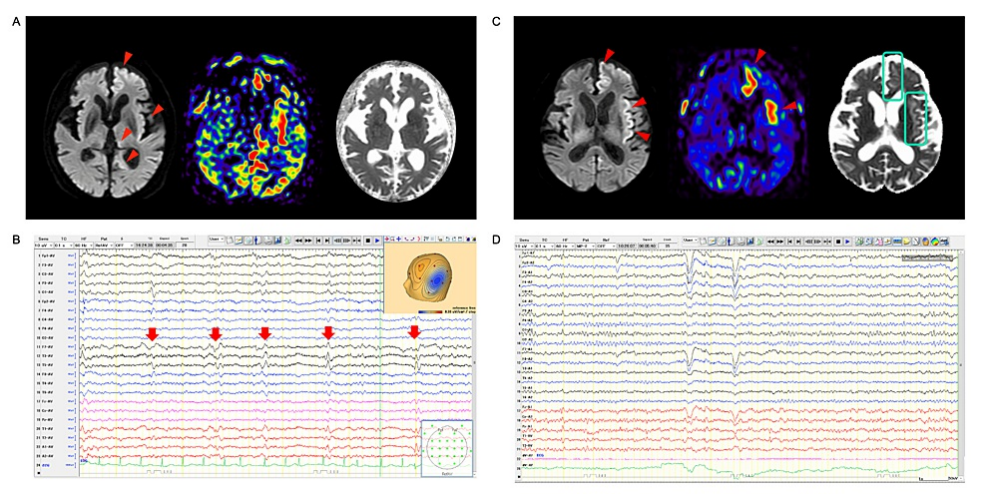
Brain MRI and EEG following initial medication A. Brain MRI shows multiple high-intensity cortical regions, including in the left hippocampus and thalamus (red arrowhead), along with hyperperfusion findings in ASL on admission, suggesting a diagnosis of status epilepticus. B. EEG on the average montage depicts <1 Hz lateralized periodic discharges on the left temporal region (red arrows) one day after the initial antiseizure medication. C. Cortical abnormal ASL findings remained only in the cortices with a “DWI-ADC match,” which is the region exhibiting cytotoxic edema, as shown in low intensity with ADC map (blue square). D. Normal background activity without epileptiform discharge is shown on day 10. ASL, arterial spin labeling; DWI-ADC, diffusion-weighted imaging-apparent diffusion coefficient; EEG, electroencephalography

A man in his 70s was admitted for acute impaired consciousness and aphasia. As he lived alone, the onset and duration of his unconsciousness remained unknown. Additionally, he had been diagnosed with mild alcoholic dementia by a physician at another hospital (based on a history of alcohol consumption and cerebral atrophy on brain CT). He had no history of epilepsy and any other initial precipitating injury, including traumatic brain injury. His vital signs were stable. On examination, he showed no external injuries. Neurological findings revealed mild impairment of consciousness and aphasia but no paresis. The patient's Glasgow Coma Scale score was 7 (E2V1M4). Blood and cerebrospinal fluid examinations revealed unremarkable findings (cerebrospinal fluid opening pressure by lumber puncture was 120 mmH2O). Brain MRI displayed multiple DWI (diffusion-weighted MRI) high-signal cortical regions, including the left hippocampus and thalamus, along with hyperperfusion findings in ASL (atrial spin labeling) (Figure 1A), suggesting a diagnosis of status epilepticus. Following the intravenous administration of diazepam (5 mg) and levetiracetam (1,000 mg), the aphasia gradually improved, and EEG revealed <1 Hz lateralized periodic discharges on the day following the initial brain MRI, indicating that the seizure activity had nearly ceased (Figure 1B). However, on day 9, in the process of gradual recovery, abnormal cortical ASL findings persisted in some cortices (Figure 1C). Conversely, follow-up EEG showed that periodic discharges had disappeared (Figure 1D). Persisted hyperperfusion findings were only visible in which low intensity in the ADC (apparent diffusion coefficient) map was evident, namely, cytotoxic edema. Without additional anti-seizure medication, the patient exhibited gradual recovery, and the seizure did not recur. He was transferred to a rehabilitation hospital with the continued oral administration of levetiracetam at 1,000 mg/day.

## Discussion

EEG recording during or immediately after seizures is vital for diagnosis or managing status epilepticus [4]. Additionally, EEG findings can also be used to ascertain risk assessment of secondary brain injury [5]. However, there are limitations in assessing neurological prognosis based on EEG alone. Thus, EEG and brain imaging findings are essential. Besides, findings of hyperperfusion in ASL are helpful in screening for epileptic seizures. However, in our case, hyperperfusion sign on ASL imaging persisted despite EEG findings confirming the clinical and electroencephalographic absence of seizure (lateralized periodic discharges of <1 Hz suggest that the patient was entering the interictal phase) [6]. Thus, if the treatment response had been determined solely by ASL without EEG, it could have led to overtreatment. Additionally, the prolonged hyperperfusion findings were visible only in the “DWI-ADC matched” region, i.e., cytotoxic edema, as shown by a high signal on DWI and a low signal on the ADC map [7]. This is the region where secondary brain injury had already occurred. Therefore, we presumably consider that no additional treatment should be given for such findings, even if hyperperfusion is visible in ASL. "DWI-ADC matched" may suggest that the therapeutic time window has ended. Thus, caution should be exercised when relying solely on ASL without evaluating EEG and assessing secondary brain injury.

## Conclusions

In order to assess the course of the status epilepticus, EEG findings as well as MRI should be evaluated on an as-needed basis. However, we presumably consider that in patients exhibiting "DWI-ADC matched" findings, careful consideration should be given to the necessity of additional ASM treatment, even if hyperperfusion is confirmed by ASL. This finding may suggest that the therapeutic time window has ended. Thus, caution should be exercised when relying solely on ASL without evaluating EEG and assessing secondary brain injury.
